# Supplementary material for: “There Is a Cat on Our Ward”: Inpatient and Staff Member Attitudes toward and Experiences with Cats in a Psychiatric Ward
Source: Int J Environ Res Public Health. 2019 Aug 27;16(17):3108. doi: 10.3390/ijerph16173108 (PMC6747524; doi:10.3390/ijerph16173108)
Supplement: Supplementary file 1 [file ijerph-16-03108-s001.pdf]

Universität Basel

Inpatients on Wards with Cats:

Date:___________________________

Ward:: ___________________________

Patientcode: _____________________

1. Gender:
   - o Male
   - o Female

2. How long have you been on this ward?

3. Did the hospitalization happen on your own request?
   - o Yes
   - o No

4. How often do you spend percentage-wise in the following areas during your therapy-free period? (Distribute 100% to the following three areas).
   - o In your room
   - o On the ward
   - o Outside the ward

5. What do you do in your spare time when you don't have therapy?

   ____
   ____
   ____
   ____
   ____

6. What are positive aspects of your ward?

   ____
   ____
   ____
   ____
   ____

7. Are you satisfied with your ward?

1 ——|——|——|——|——|—— 6

Not at all satisfied — very satisfied

8. What is important to you regarding ward atmosphere?

____

____

____

____

9. Do you know that a cat lives on your ward?
   - o Yes
   - o No

10. How often do you see the cat?

1 — | — | — | — | — 6

never ... very often

11. Is the cat trustful?
   - o Yes
   - o No

12. How close is your contact to the cat?

1 — | — | — | — | — 6

Not at all close ... very close

13. How is your emotional relation to the cat?

1 — | — | — | — | — 6

negative ... positive

14. How much time do you spend with the cat daily?
   - o No time
   - o Up to 10 minutes
   - o Up to 30 minutes
   - o Up to 60 minutes
   - o More than 60 minutes

15. Do you search the ward cat actively?
   - o Yes
   - o No

16. How do you feel about housing a ward cat?

1 — 6

negative — positive

a) What are positive aspects?

____
____
____
____
____

b) What are negative aspects?

____
____
____
____
____

17. How does the cat influence your stay at the clinic?

1 — 6

negative — positive

18. How does the cat influnece your emotional well-being?

1 — 6

negative — positive

19. Does the cat help you to feel more comfortable on your ward?

- Yes
- No

20. How would the ward atmosphere change if the ward cat was no longer there?

1 — 6

negative — positive

21. Does the ward cat lead to more conversation between you and other inpatients?

- Yes
- No

22. Would you prefer it the cat would not be living on your ward any more?
    - o Yes
    - o No

a) Why?

____

23. Would you prefer another animal?
    - o Yes
    - o No

a) If yes, what animal:

____

24. Did you grow up with animals?
    - o Yes
    - o No

25. Did you have pets?
    - o Yes
    - o No

a) If yes, what kind of pets:

____

26. Do you have pets at the moment?
    - o Ja
    - o Nein

a) If yes, what kind of pets:

____

27. How were your experiences with pets?

1 |—|—|—|—|—| 6

negative … positive

28. Do you like animals?

1 |—|—|—|—|—| 6

Not at all … very much

29. Do you like cats?

1 |—|—|—|—|—| 6

Not at all … very much

30. Do you have a cathair allergy?
    - o Yes
    - o No

31. Comments:

Staff on Wards with Cats

Date: ____________________________

Ward: ____________________________

Code: ______________________

32. Gender
    - o Male
    - o Female

33. How long have you been working on this ward?

    ____

34. Are you satisfied with your ward?

    1 — — — — — 6

    Not at all satisfied ... very satisfied

35. What is important to you regarding ward atmosphere?

    ____
    ____
    ____
    ____
    ____

36. Do you know that there is a cat living on your ward?
    - o Yes
    - o No

37. How often du you see the cat?

    1 — — — — — 6

    Never ... very often

38. Is the cat trustful?
    - o Yes
    - o No

39. How close is your contact to the cat?

1 — 6

Not at all close — very close

40. How is your emotional relation to the cat?

1 — 6

negative — positive

41. How much time do you spend with the cat daily?

1 — 6

Not at all much — very much

42. Does the ward cat lead to an additional effort for staff members?

- Yes
- No

43. Who takes care of the cat?

- Staff member
- Patient

44. How do you feel about that there is a cat living on your ward?

1 — 6

negative — positive

a) What are positive aspects?

____

____

____

____

____

b) What are negative aspects?

____

____

____

____

____

45. How does the ward cat influence your work satisfaction?

1 — 6

negative — positive

46. How does the ward cat influence ward atmosphere?

1 — 6

negative — positive

47. How would the ward atmosphere change if the cat would not be living on the ward anymore?

1 — 6

negative — positive

48. Do you believe that the ward cat helps inpatients to feel more comfortable on the ward?
   - o Yes
   - o No

49. Do you believe that the ward cat helps to make the ward more cozy?
   - o Yes
   - o No

50. How does the ward cat influence inpatients well-being?

1 — 6

negative — positive

51. Does the ward cat lead to more conversation between inpatients?
   - o Yes
   - o No

52. Does the ward cat lead to more conversation between inpatients and staff members?
   - o Yes
   - o No

53. Would you prefer if the ward cat would not be living on your ward anymore?
   - o Yes
   - o No

a) If yes, why:

____

54. Would you prefer another animal?
   - o Yes

- No

a) If yes, what kind of animal:

____

55. Did you grow up with animals?
    - Yes
    - No

56. Did you have pets?
    - Yes
    - No

a) If yes, what kind of pets:

____

57. Do you have pets at the moment?
    - Yes
    - No

a) If yes, what kind of pets:

____

58. Did you have positive experiences with pets?
    - Yes
    - No

59. Do you like animals?

1 — 6

Not at all … very much

60. Do you like cats?

1 — 6

Not at all … very much

61. Do you have a cathair allergy?
    - Yes
    - No

62. Comments:

____

____

____

____

____

Universität Basel

Inpatients on Wards without Cats

Date: ________________________

Ward: ________________________

Patientcode: ____________________

63. Gender:
    - o Male
    - o Female

64. How long have you been on this ward?

    ____

65. Did the hospitalization happen on your own request?
    - o Yes
    - o No

66. How often do you spend percentage-wise in the following areas during your therapy-free period? (Distribute 100% to the following three areas).
    - o In your room
    - o On the ward
    - o Outside the ward

67. What do you do in your spare time when you don't have therapy?

    ____
    ____
    ____
    ____
    ____

68. What are positive aspects of your ward?

    ____
    ____
    ____
    ____
    ____

69. What is important to you regarding ward atmosphere?

    ____
    ____
    ____
    ____
    ____

70. How satisfied are you with your ward?

1 ——— ——— ——— ——— ——— 6

Not at all satisfied — Very satisfied

71. Do you know that some wards are housing cats?
   - Yes
   - No

72. How do you feel about that some wards are housing a cat?

1 ——— ——— ——— ——— ——— 6

negative — positive

c) What do you think are positive aspects of housing a cat on a ward?

____

____

____

____

____

d) What do you think are negative aspects of housing a cat on a ward?

____

____

____

____

____

73. How could a ward cat influence the ward atmosphere?

1 ——— ——— ——— ——— ——— 6

negative — positive

74. How could a ward cat influence your satisfaction with the ward?

1 ——— ——— ——— ——— ——— 6

negative — positive

75. Do you think that a ward cat could help you to feel more comfortable on your ward?

1 ——— ——— ——— ——— ——— 6

Not at all — very

76. Would you prefer to have a cat on your ward?
   - o Yes
   - o No

   a) Why?

   ____

77. Would you prefer another ward animal than a cat?
   - o Yes
   - o No

a) If yes, which:

____

78. Did you grow up with animals?
   - o Yes
   - o No

79. Did you have pets?
   - o Yes
   - o No

a) If yes, what kind of pets:

____

80. Do you have pets at the moment?
   - o Yes
   - o No

a) If yes, what kind of pets:

____

81. How were your experiences with pets?

1 — | — | — | — | — 6

negative … positive

82. Do you like animals?

1 — | — | — | — | — 6

Not at all … very much

Universität Basel

83. Do you like cats?

1 — — — — — 6

Not at all — very much

84. Do you have a cathair allergy?
    - Yes
    - No

85. Comments:
